# Extremely low light promotes adventitious shoot regeneration via RpPHYA/RpHYH-antagonized auxin biosynthesis and transport through dual regulation of *RpPIN1* in *Robinia pseudoacacia*

**DOI:** 10.1093/hr/uhag202

**Published:** 2026-05-25

**Authors:** Juan Han, Yanting Tian, Jie Liu, Houyin Deng, Kunjin Han, Huayu Si, Shasha Ling, Shumin Zhang, Lixin Yu, Yousry A El-Kassaby, Yuhan Sun, Zuodeng Peng, Yun Li, Ye Zhao

**Affiliations:** State Key Laboratory of Tree Genetics and Breeding, Engineering Technology Research Center of Black Locust of National Forestry and Grassland Administration, College of Biological Sciences and Technology, Beijing Forestry University, Beijing 100083, China; The Key Laboratory for Silviculture and Conservation of Ministry of Education, College of Forestry, Beijing Forestry University, Beijing 100083, China; State Key Laboratory of Tree Genetics and Breeding, Engineering Technology Research Center of Black Locust of National Forestry and Grassland Administration, College of Biological Sciences and Technology, Beijing Forestry University, Beijing 100083, China; The Research Institute of Forestry and Pomology, Tianjin Academy of Agricultural Sciences, Tianjin 300192, China; State Key Laboratory of Tree Genetics and Breeding, Engineering Technology Research Center of Black Locust of National Forestry and Grassland Administration, College of Biological Sciences and Technology, Beijing Forestry University, Beijing 100083, China; State Key Laboratory of Tree Genetics and Breeding, Engineering Technology Research Center of Black Locust of National Forestry and Grassland Administration, College of Biological Sciences and Technology, Beijing Forestry University, Beijing 100083, China; State Key Laboratory of Tree Genetics and Breeding, Engineering Technology Research Center of Black Locust of National Forestry and Grassland Administration, College of Biological Sciences and Technology, Beijing Forestry University, Beijing 100083, China; State Key Laboratory of Tree Genetics and Breeding, Engineering Technology Research Center of Black Locust of National Forestry and Grassland Administration, College of Biological Sciences and Technology, Beijing Forestry University, Beijing 100083, China; State Key Laboratory of Tree Genetics and Breeding, Engineering Technology Research Center of Black Locust of National Forestry and Grassland Administration, College of Biological Sciences and Technology, Beijing Forestry University, Beijing 100083, China; State-Owned Huangtuliangzi Forestry Station in Pingquan City of Hebei Province, Pingquan 067500, China; State-Owned Huangtuliangzi Forestry Station in Pingquan City of Hebei Province, Pingquan 067500, China; Department of Forest and Conservation Sciences, Faculty of Forestry and Environmental Stewardship, The University of British Columbia, 2424 Main Mall, Vancouver, BC V6T 1Z4, Canada; State Key Laboratory of Tree Genetics and Breeding, Engineering Technology Research Center of Black Locust of National Forestry and Grassland Administration, College of Biological Sciences and Technology, Beijing Forestry University, Beijing 100083, China; The Key Laboratory for Silviculture and Conservation of Ministry of Education, College of Forestry, Beijing Forestry University, Beijing 100083, China; State Key Laboratory of Tree Genetics and Breeding, Engineering Technology Research Center of Black Locust of National Forestry and Grassland Administration, College of Biological Sciences and Technology, Beijing Forestry University, Beijing 100083, China; State Key Laboratory of Tree Genetics and Breeding, Engineering Technology Research Center of Black Locust of National Forestry and Grassland Administration, College of Biological Sciences and Technology, Beijing Forestry University, Beijing 100083, China

## Abstract

Extremely low light (ELL) is widely applied as a strategy to enhance adventitious shoot (AS) regeneration in woody plants. However, how ELL acts as an environmental signal to reprogram regenerative competence remains unclear. Here, we show that ELL for 14 days (ELL14) markedly promotes AS regeneration across multiple genotypes of *Robinia pseudoacacia*. Transcriptomic and functional enrichment analyses revealed that auxin biosynthesis, transport, and signaling constitute the core pathways activated under ELL14. Consistently, auxin levels increased, and the inhibition of biosynthesis or transport largely abolished the regeneration-promoting effect. Integrative weighted gene co-expression network analysis and regulatory analyses identified RpPHYA as a key integrator of ELL-responsive networks. Under ELL14, the RpPHYA-associated module promotes auxin biosynthesis by upregulating *RpAMI1* and coordinates auxin transport through *RpPIN1* via transcriptional regulation and *RpROS1*-mediated CHG demethylation, forming a dual regulatory mechanism. In contrast, under normal light, RpHYH antagonizes this regulatory program, thereby restricting regenerative capacity. Overexpression of *RpPHYA*, *RpAMI1*, *RpPIN1*, and *RpROS1* significantly enhanced AS regeneration, functionally validating this ELL14-induced module. Collectively, our findings demonstrate that ELL functions as an environmental signal that promotes AS regeneration in *R. pseudoacacia* through coordinated transcriptional and DNA demethylation of auxin transport, providing mechanistic insights for improving regeneration in woody plants.

## Introduction

Adventitious shoot (AS) regeneration, a central pathway of organogenesis, underlies efficient clonal propagation, genetic transformation, and genome editing [1], and is broadly influenced by light conditions [2]. Notably, the effects of light conditions on AS regeneration vary markedly among species, genotypes, and explant types. For example, high-intensity light enhances AS regeneration from root explants [3], whereas extremely low light (ELL) has been reported to enhance AS formation from cotyledon explants in *Arabidopsis* [4]. In woody plants, low light has been applied as a practical strategy to enhance AS regeneration efficiency [5]. For example, reducing light intensity from 2000–2500 l**×** to 50–100 l**×** markedly increased the regeneration efficiency of leaf explants from 6.3 ± 3.8% to 72.5 ± 4.8% in *Platanus acerifolia* [6]. Similarly, ELL increased callus induction from leaf explants to 85.6% in *Punica granatum* ‘Taishan Red’, thereby facilitating subsequent AS regeneration [7]. Collectively, ELL induced by these treatments are widely used to improve regeneration efficiency in woody plants. However, the molecular mechanisms by which ELL enhance AS regeneration are still poorly understood. This gap remains an important question in plant regeneration biology.

Studies on normal light-regulated AS regeneration can provide valuable insights into how ELL signals influence regenerative processes. For example, in tomato leaf explants, normal light promotes AS regeneration by enhancing photosynthetic activity [8]. In addition to photosynthesis, light also modulates AS regeneration through multiple regulatory pathways. For example, ELONGATED HYPOCOTYL 5 (HY5) represses shoot meristem and hormone-related genes, including *WUSCHEL* (*AtWUS*), *CLAVATA3* (*AtCLV3*), and *ARABIDOPSIS RESPONSE REGULATOR 12* (*AtARR12*), thereby reducing AS regeneration [9–12]. In addition, epigenetic regulation also plays an important role. For example, *met1* mutants show decreased CG methylation at *CRYPTOCHROME 1/2* (*CRY1/2*), which enhances their expression and promotes ARR1-mediated shoot formation in *Arabidopsis* [13]. Together, these observations suggest that ELL may also regulate AS regeneration through complex crosstalk with hormonal and epigenetic pathways. However, the upstream mechanisms that integrate ELL signals with hormonal and epigenetic regulation remain largely unknown. Among photoreceptors, phytochrome A (PHYA) functions as a primary sensor of low light and far-red-enriched light environments [14, 15]. In *Arabidopsis*, AtPHYA has been shown to modulate auxin signaling by interacting with AUX/IAA proteins and altering their stability [16]. However, whether and how PHYA coordinates both hormonal and epigenetic regulation to modulate AS regeneration under ELL remains unclear.

Auxin, a central regulator of AS regeneration, is not only essential for directing cell fate reprogramming within explants but is also dynamically modulated by environmental cues [17–20]. The indole-3-pyruvic acid (IPA) and indole-3-acetamide (IAM) pathways play prominent roles in auxin biosynthesis [21]. The IPA pathway, catalyzed by YUCCA (YUC) flavin monooxygenases, is a major auxin biosynthesis route that drives local auxin accumulation during early regeneration [19, 22]. Simultaneously, the IAM pathway contributes to auxin biosynthesis through INDOLE-3-ACETAMIDE HYDROLASE 1 (AMI1), which converts IAM to indole-3-acetic acid (IAA) [23]. However, the role of AMI1 in auxin-regulated developmental processes, particularly during AS regeneration, remains relatively underexplored. Alongside auxin biosynthesis, auxin transport plays a crucial role in regeneration. The auxin efflux carriers, particularly PIN-FORMED (PIN) proteins, create auxin maxima and spatial gradients, determining the initiation and positioning of shoot primordia [24–26]. However, despite the known importance of auxin, how PIN-mediated auxin transport responds specifically to environmental cues such as ELL remains largely unexplored.

DNA methylation plays an important regulatory role in plant growth and development, with recent studies indicating its importance in AS regeneration [11, 12, 27]. In *Arabidopsis*, maintenance methyltransferases have been shown to modulate AS regeneration [28]. For instance, *met1* mutants exhibit enhanced AS regeneration, associated with the derepression of *WUS* [5, 13, 28]. In contrast, active DNA demethylases, such as REPRESSOR OF SILENCING 1 (ROS1), remove DNA methylation marks, particularly at CHG sites. This process dynamically modulates gene expression. ROS1 has been implicated in stress responses and reproductive development [29, 30], but its role in AS regeneration remains largely unexplored. DNA methylation has also been shown to influence AS regeneration by modulating light-responsive transcription factors. However, the crosstalk between these two regulatory layers remains largely unexplored.

*Robinia pseudoacacia* (black locust) is a globally significant woody species with important horticultural and economic value [31, 32]. It is prized for its diverse ornamental cultivars and fragrant, nectar-rich inflorescences. In addition, it has recently gained attention as a source of bioactive-rich edible flowers [33, 34]. To meet the increasing market demand for elite ornamental traits and enhanced nutritional quality, efficient micropropagation and genetic improvement systems are essential. However, the large-scale application of these biotechnological tools is frequently hampered by significant recalcitrance in tissue culture. Like many woody perennials, *R. pseudoacacia* exhibits strong genotype dependence and accumulates high levels of secondary metabolites, particularly phenolics. These often cause severe tissue browning and low regeneration efficiency [35–37]. These limitations make it an ideal yet challenging model to investigate the molecular mechanisms underlying ELL-mediated AS regeneration. In this study, we systematically investigated the effects of ELL on AS regeneration in *R. pseudoacacia*. Integrated analyses of morphology, histology, hormones, transcriptomes, and DNA methylation indicate that ELL promotes regeneration by coordinately modulating auxin-related transcription and DNA demethylation, thereby enhancing auxin biosynthesis and polar transport. Notably, the RpPHYA-associated regulatory module transduces ELL signals to activate *RpAMI1* and *RpPIN1* expression and promote *RpROS1*-mediated CHG demethylation at the *RpPIN1* promoter, further facilitating auxin transport. This dual regulatory mechanism provides the first direct evidence in a woody plant linking environmental ELL signals to coordinated transcriptional and DNA demethylation control of regenerative competence.

## Results

### ELL14 significantly enhances AS regeneration in *R. pseudoacacia*

We quantified light intensity under ELL and CK using a spectroradiometer and converted spectral radiance to photon flux density. The spectral distribution remained largely unchanged, indicating that ELL primarily reduced light intensity rather than CK (Fig. S1A). Total photosynthetic photon flux density (PPFD) (400–700 nm) was 1.45 μmol·m^−2^·s^−1^ under ELL compared to 86.66 μmol·m^−2^·s^−1^ under CK, representing a 98.3% reduction (Fig. S1B). Leaves were subjected to ELL preconditioning for 7, 14, or 21 days, with untreated leaves serving as CK (Fig. 1A). At 30, 40, and 50 days after culture, both the average number of AS per leaf and the regeneration frequency were significantly higher in ELL14 and ELL21 than in CK and ELL7 (Fig. 1B–D). Notably, ELL14 exhibited a regeneration performance comparable to, or slightly better than, that of ELL21. Therefore, ELL14 was selected as the representative optimal treatment for subsequent analyses, as it achieved high regeneration efficiency with a shorter treatment duration. These results were consistent across repeated experiments (Fig. S2A-B). We further assessed the effect of ELL14 treatment by measuring the expression of three AS regeneration marker genes-*RpWUS*, *WUSCHEL-RELATED HOMEOBOX 4* (*RpWOX4*), and *LATERAL ORGAN BOUNDARIES DOMAIN 41* (*RpLBD41*) [38, 39]. In ELL14-treated leaves, *RpWOX4* and *RpLBD41* was significantly upregulated at 8, 11, 14 days (Fig. 1F and G) and *RpWUS* at 8 and 14 days (Fig. 1E) compared to CK, confirming that ELL14 promotes AS regeneration in ‘PU18-22’. To determine whether this effect is genotype-dependent, the same treatments were applied to two diploid (‘P28-15’ and ‘M28-34’) and two tetraploid (‘38’ and ‘A4’) genotypes (Fig. S3). The results showed that ELL for an optimal duration enhanced AS regeneration across all four genotypes (Fig. S3A–L). We also examined the expression of three AS regeneration marker genes (*RpWUS*, *RpWOX4*, and *RpLBD41*) [40, 41] across all four genotypes (Fig. S4). In each genotype, the expression levels of these genes were consistently higher under ELL14 treatment than under CK treatment at key time point (Fig. S4A–D). Collectively, these results demonstrate that ELL14 universally enhances AS regeneration across different genotypes of *R. pseudoacacia*.

**Figure 1:**
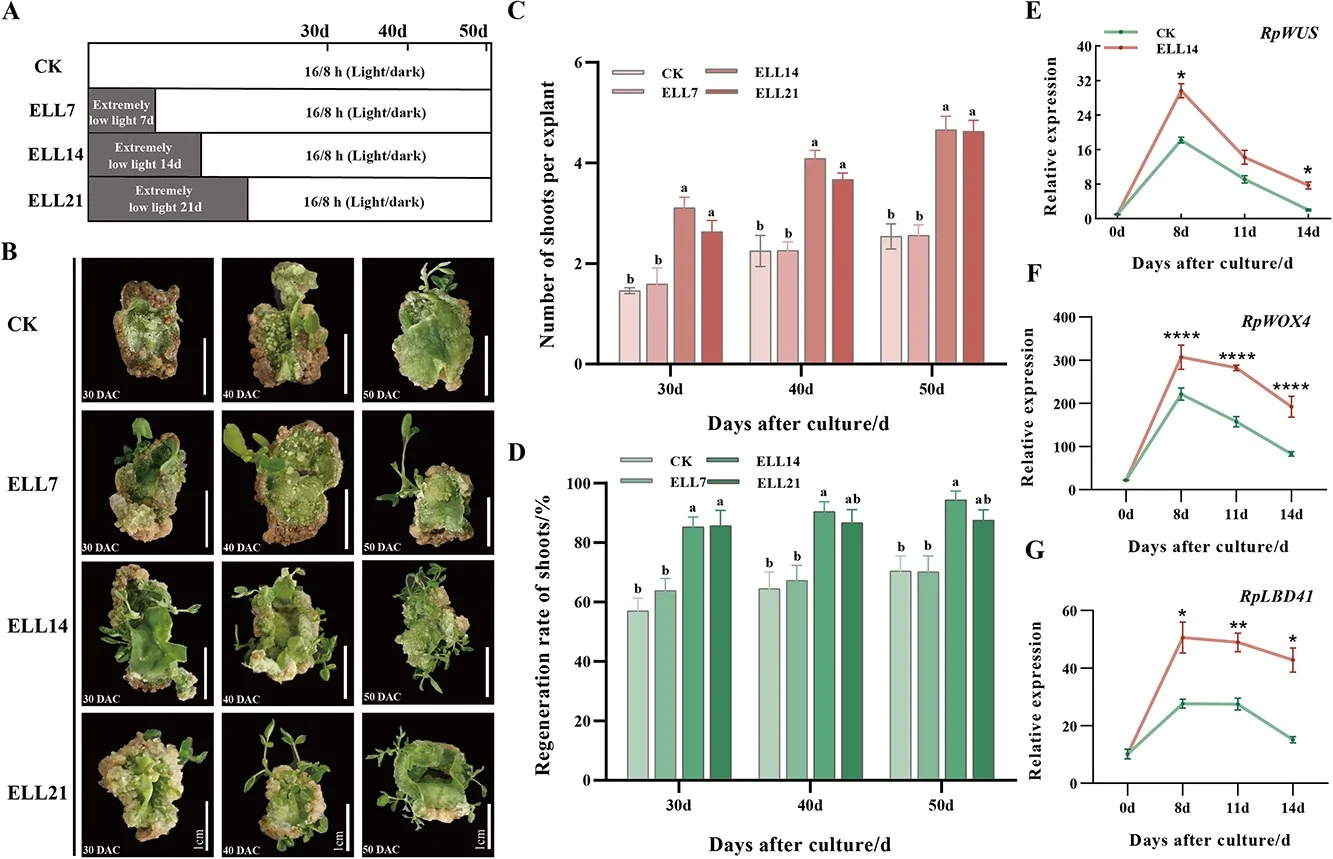
Effects of CK, ELL7, ELL14, and ELL21 on AS regeneration and the expression of key regeneration-related genes (*RpWUS*, *RpWOX4*, and *RpLBD41*) in *R. pseudoacacia* leaf explants of genotypes ‘PU18-22’ under CK and ELL14. (A) Schematic diagram showing the ELL treatment, the time points for AS regeneration data collection. (B) Phenotypic observation of AS regeneration from leaf explants of genotype ‘PU18-22’ under CK and different ELL durations. DAC, days after culture. (C) Average number of AS and regeneration efficiency of ‘PU18-22’ leaf explants at 30, 40, and 50 days under CK and different ELL durations. (D) Regeneration efficiency of AS from ‘PU18-22’ leaf explants at 30, 40, and 50 days under CK and different ELL durations. (E–G) Expression analysis of *RpWUS*, *RpWOX4*, and *RpLBD41* during AS regeneration from leaf explants of ‘PU18-22’ under CK and ELL14. Statistical significance is indicated as follows: *P* < 0.05, *P* < 0.01, *P* < 0.001, *P* < 0.0001.

### ELL14 accelerates AS formation in ‘PU18-22’ leaves

As ELL14-mediated enhancement of AS regeneration was independent of genotype, the vigorously growing genotype ‘PU18-22’ was chosen for further mechanistic analysis. Leaves from the CK and ELL14 treatments during the first 14 days were observed (Fig. 2A–D). The leaves showed folding and enlargement, and by day 14, shoot primordia were visible on the abaxial surface of leaves in the ELL14 treatment (Fig. 2A). Subsequently, histological sections were analyzed. At Day 8, active cell division was observed in both treatments (Fig. 2B). In ELL14-treated leaves, proliferating internal cells had already extended to the lower epidermis and showed signs of integrity loss. In contrast, the lower epidermis in CK-treated leaves remained intact (Fig. 2B). By Day 11, distinct shoot primordia were visible in ELL14-treated leaves, while CK-treated leaves were still undergoing active cell division. At Day 14, well-developed shoot primordia were observed in ELL14-treated leaves, whereas CK-treated leaves had only just begun to form visible shoot primordia (Fig. 2B). SEM observations were consistent with the results of semi-thin section, further confirming that ELL14 treatment accelerates AS regeneration in *R. pseudoacacia* leaves (Fig. 2C). To further validate these findings, we performed *in situ* hybridization using *RpWUS* probe (Fig. 2D). At Day 11, strong hybridization signals of RpWUS were detected in the emerging shoot primordia of ELL14-treated leaves, whereas only weak signals were observed in CK-treated leaves. By Day 14, *RpWUS* expression was broadly distributed around well-formed shoot primordia in ELL14-treated leaves, whereas CK-treated leaves showed clear *RpWUS* expression only in newly formed shoot primordia (Fig. 2D). Collectively, these results indicate that ELL14 substantially accelerates the formation of shoot primordia in *R. pseudoacacia* leaf explants.

**Figure 2:**
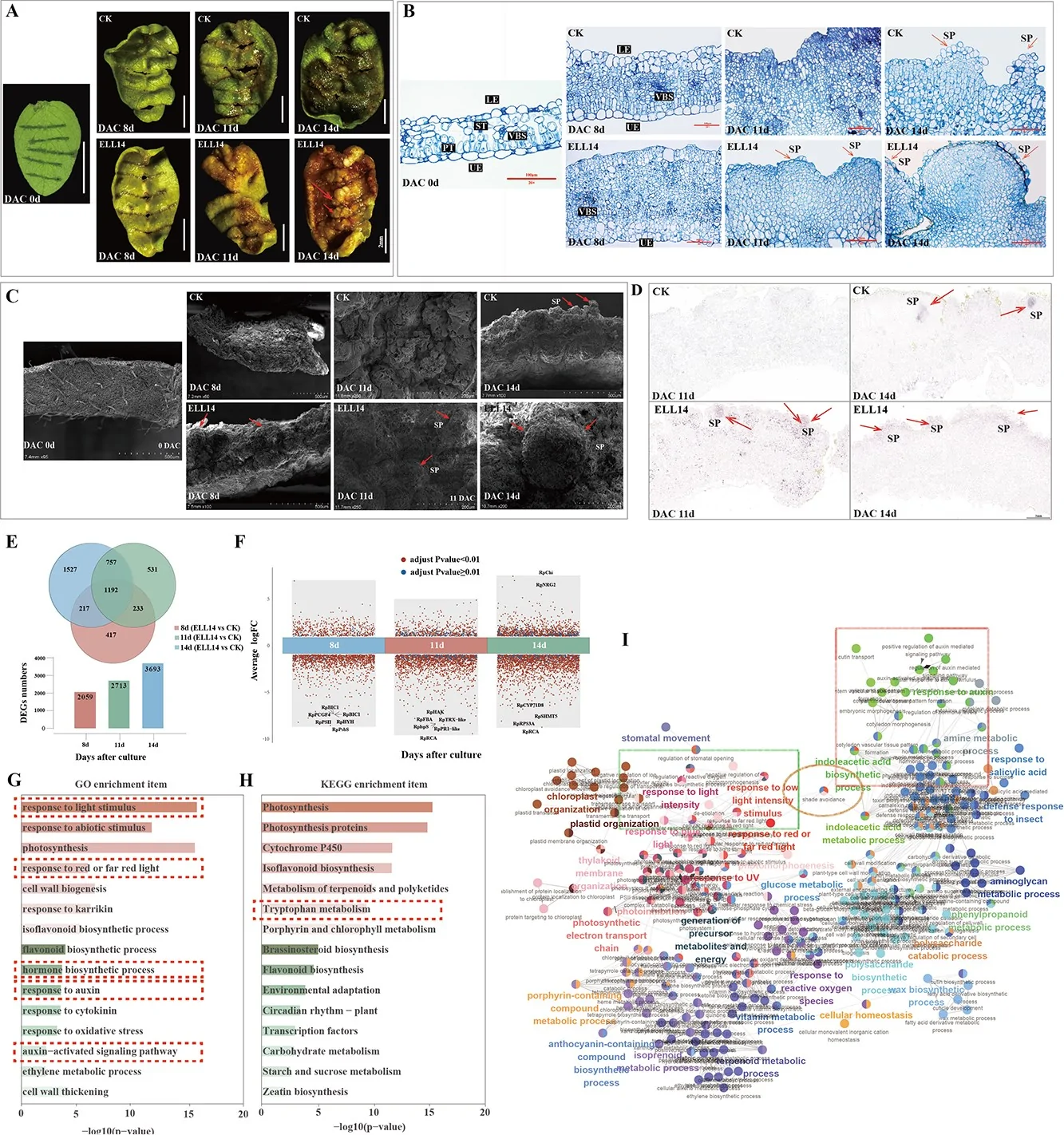
Morphological, histological, and transcriptomic analyses of AS regeneration from leaf explants of ‘PU18-22’ under CK and ELL14 treatments. (A) Morphological observation of leaf explants at 8, 11, and 14 days during AS regeneration under CK and ELL14. DAC, days after culture. (B) Histological section analysis of leaf explants at 8, 11, and 14 days during AS regeneration under CK and ELL14 (LE, lower epidermis; UE, upper epidermis; VBS, vascular bundle sheath; ST, stomata; PT, palisade tissue). Arrows indicate shoot primordia (SP). (C) SEM observation of leaf explants at 8, 11, and 14 days during AS regeneration under CK and ELL14. Arrows indicate shoot primordia (SP). (D) *In situ* hybridization analysis of *RpWUS* expression in leaf explants at 11 and 14 days during AS regeneration under CK and ELL14. (E) Number of DEGs identified at 8, 11, and 14 days during AS regeneration under CK and ELL14. (F) Visualization of DEGs identified at 8, 11, and 14 days during AS regeneration under CK and ELL14. (G) GO enrichment analysis of the overlapping DEGs identified at 8, 11, and 14 days under CK and ELL14. (H) KEGG pathway enrichment analysis of the overlapping DEGs identified at 8, 11, and 14 days under CK and ELL14. (I) ClueGO analysis of enriched pathways based on DEGs identified at 8, 11, and 14 days during AS regeneration under CK and ELL14.

### Auxin-related pathways are significantly enriched during ELL14

To investigate the molecular mechanism by which ELL14 treatment promotes AS regeneration in *R. pseudoacacia* leaves, RNA-seq was performed using samples collected at 8, 11, and 14 days under CK and ELL14 treatments. Correlation and principal component analysis analyses, together with RT-qPCR validation of key hormone- and regeneration-related genes such as AUXIN RESPONSE FACTOR 10 (*RpARF10*), *RpWUS*, and ARABIDOPSIS RESPONSE REGULATOR 1 (*RpARR1*), collectively confirmed the high reliability of the RNA-seq data (Fig. S5A–C). We identified 2059, 2713, and 3693 differentially expressed genes (DEGs) at 8, 11, and 14 days, respectively (Fig. 2E; Table S1–S6). Visualization of DEGs revealed that most downregulated genes were associated with responses to light stimulus and photosynthesis (Fig. 2F). Gene Ontology (GO) and Kyoto Encyclopedia of Genes and Genomes (KEGG) enrichment were performed using the union of DEGs from all three time points (4875 genes; Fig. 2G and H). The results showed that these DEGs were mainly enriched in pathways associated with light response, hormone biosynthesis, auxin response, auxin signaling, and tryptophan (Trp) metabolism, suggesting a potential role for auxin in ELL14-mediated AS regeneration. To explore the potential relationships among these enriched GO terms, a ClueGO network analysis was performed (Fig. 2I). The results showed that the term ‘response to light stimulus’ was connected to both ‘indoleacetic acid biosynthetic process’ and ‘response to auxin’ in the network through ‘shade avoidance’. This suggests that the ELL signaling pathway may regulate AS regeneration by modulating auxin-related pathways.

### ELL14 enhances AS regeneration by promoting auxin biosynthesis and transport

Considering the enrichment of DEGs in auxin-related pathways and the potential regulation of auxin by ELL14, we measured auxin levels in CK- and ELL14-treated leaves (Fig. 3A). The levels of IAA, Trp, and indole-3-acetonitrile (IAN) were consistently higher in ELL14-treated leaves than in CK at 8, 11, and 14 days, while indole-3-butyric acid (IBA) levels were significantly increased at 11 and 14 days (Fig. 3A–D). These findings indicate that auxin biosynthesis is strongly induced under ELL14, which likely promotes AS formation. To determine which auxin-related pathways are involved in ELL14, the auxin biosynthesis inhibitor yucasin (yuc) and the auxin transport inhibitor NPA were applied under both CK and ELL14 conditions. AS regeneration was then assessed 40 days after inoculation (Fig. 3E–H). We found that after treatment with the auxin biosynthesis inhibitor yuc, no significant difference in AS regeneration efficiency was observed between ELL14 and CK. In addition, the difference in regenerated shoot number was markedly reduced (Fig. 3E and F). Similarly, treatment with the auxin polar transport inhibitor NPA significantly diminished the differences in both AS regeneration efficiency and shoot number between CK and ELL14 (Fig. 3G and H). This indicates that auxin biosynthesis and polar transport are involved in the promotive effect of ELL14 on AS regeneration.

**Figure 3:**
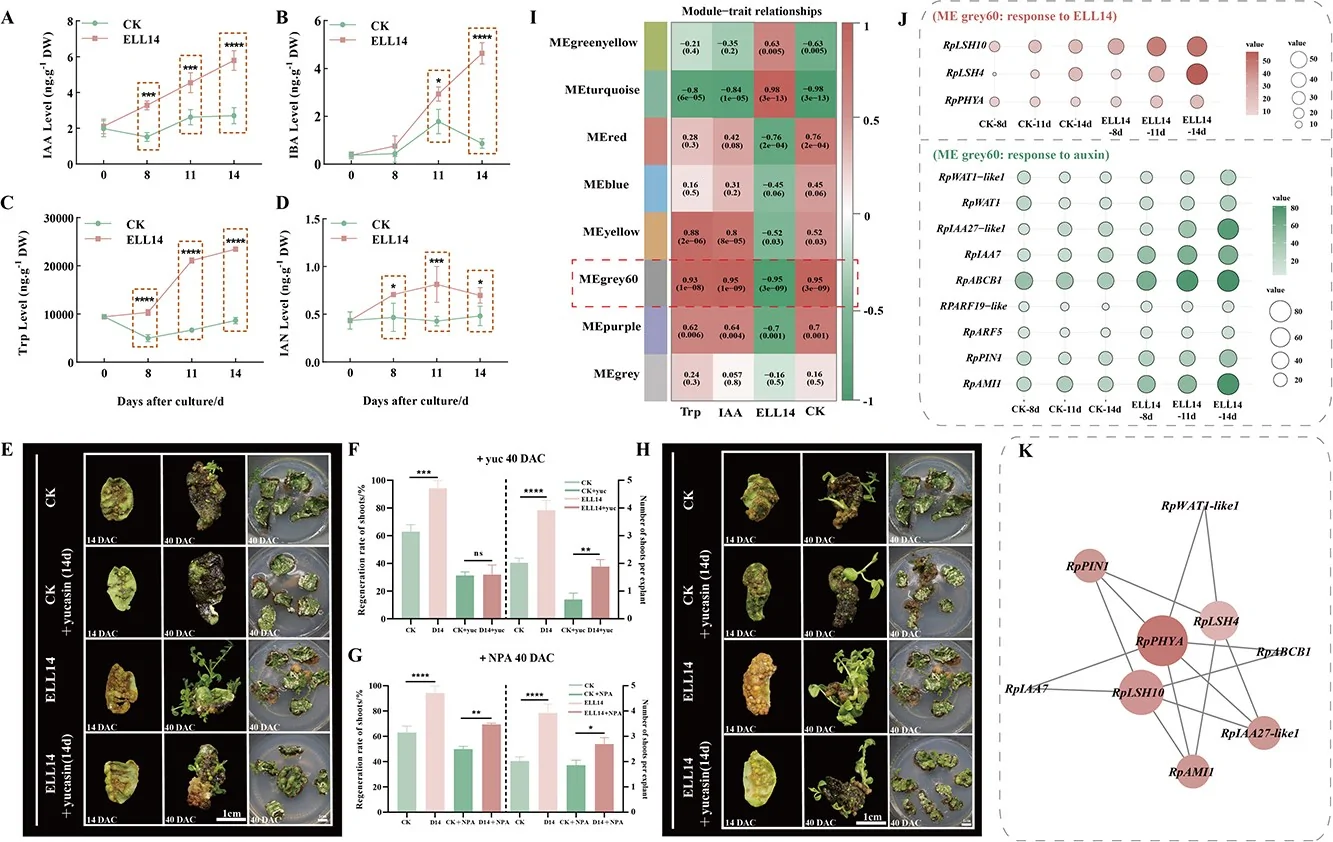
ELL14 enhances auxin accumulation and reprograms auxin-associated transcriptional modules during AS regeneration. (A) Changes in IAA content during AS regeneration from leaf explants of ‘PU18-22’ under CK and ELL14. (B) Changes in IBA content during AS regeneration from leaf explants of ‘PU18-22’ under CK and ELL14. (C) Changes in Trp content during AS regeneration from leaf explants of ‘PU18-22’ under CK and ELL14. (D) Changes in IAN content during AS regeneration from leaf explants of ‘PU18-22’ under CK and ELL14. (E and F) Statistical analysis of AS shoot regeneration from ‘PU18-22’ leaf explants at 40 days following treatment with the auxin biosynthesis inhibitor yucasin under CK and ELL14. DAC, days after culture. (G and H) Statistical analysis of AS regeneration from ‘PU18-22’ leaf explants at 40 days following treatment with the auxin transport inhibitor NPA under CK and ELL14. (I) WGCNA of the overlapping DEGs identified at 8, 11, and 14 days in relation to Trp content, IAA content, and light conditions. (J) Visualization of ELL14-responsive DEGs and auxin-responsive DEGs in the MEgrey60 modules. (K) Correlation network analysis of key genes responsive to ELL14 and auxin signaling in the MEgrey60 modules. Statistical significance is indicated as follows: *P* < 0.05, *P* < 0.01, *P* < 0.001, *P* < 0.0001.

### Co-expression network reveals interactions among genes involved in auxin biosynthesis and transport under ELL14

To identify the genes underlying auxin biosynthesis and transport in ELL14-enhanced AS regeneration, weighted gene co-expression network analysis (WGCNA) was performed to correlate Trp, IAA, and ELL14 with DEGs across three time points (Fig. 3I; Table S4). The analysis revealed that the module ‘Megrey60’ was highly and positively correlated with Trp, IAA levels, and ELL14 (Fig. 3I). Expression of MEgrey60 module genes responsive to ELL14 and auxin was visualized (Fig. 3J). These included the low-light-responsive gene *RpPHYA*, as well as genes involved in auxin biosynthesis and polar transport, such as *RpAMI1* and *RpPIN1*, all of which were significantly upregulated following ELL14 compared to CK (Fig. 3J and S6). Pearson correlation analysis was applied to these genes, and strongly correlated genes were used to construct a co-expression network, revealing key gene-to-gene interactions (Fig. 3K; Table S5). The results showed that *RpPHYA* occupied a central position in the network and was connected to the auxin biosynthesis and transport genes *RpAMI1* and *RpPIN1* (Fig. 3K and S6B), suggesting that these genes may be involved in AS regeneration under ELL14. The expression patterns of *RpAMI1* and *RpPIN1* were examined during AS regeneration under CK and ELL14 treatments in four additional genotypes: ‘P28-15’ (Fig. S7A), ‘M28-34’ (Fig. S7B), ‘38’ (Fig. S7B and C), and ‘A4’ (Fig. S7D). The results showed that, at different developmental stages, *RpAMI1* and *RpPIN1* expression levels were consistently higher under ELL14 than CK. These findings further confirm that *RpAMI1* and *RpPIN1* are broadly involved in ELL14-mediated AS regeneration.

### RpPHYA-associated module activates *RpAMI1* and *RpPIN1* expression under ELL14, whereas RpHYH represses it under CK

Previous studies have shown that PHYA regulates gene expression through transcription factor-associated complexes, and that G-box elements are key *cis*-regulatory motifs involved in PHYA-mediated transcriptional regulation [42]. Consistent with the strong co-expression of *RpPHYA* with *RpAMI1* and *RpPIN1*, promoter analysis revealed four G-box elements in the *RpAMI1* promoter and one G-box together with one GT-1 element in the *RpPIN1* promoter (Fig. 4A), suggesting that both genes may be transcriptionally regulated by an RpPHYA-associated regulatory module. To test this hypothesis, luciferase (LUC) assays were performed, showing that the RpPHYA-associated module significantly enhanced the promoter activities of *RpAMI1* and *RpPIN1* compared to the control (Fig. 4B). To further assess the role of G-box elements, mutation-based LUC assays were performed. Mutation of the key G-box motifs in the promoters of *RpAMI1* and *RpPIN1* markedly reduced the activation effect of the RpPHYA-associated module (Fig. S8A and B), indicating that this regulation depends on G-box *cis*-elements. Yeast one-hybrid (Y1H) assays (*in vivo*) and electrophoretic mobility shift assay (EMSA) assays (*in vitro*) were conducted. In the Y1H system, the RpPHYA was only able to weakly activate reporter constructs containing specific regions of the *RpAMI1* and *RpPIN1* promoters (Fig. S8C and D). EMSA analysis showed that RpPHYA protein alone could not stably bind to the *RpAMI1* promoter fragments (Fig. S8E–H). These results suggest that RpPHYA is unlikely to function through direct DNA binding, but rather may depend on other factors to regulate transcription.

**Figure 4:**
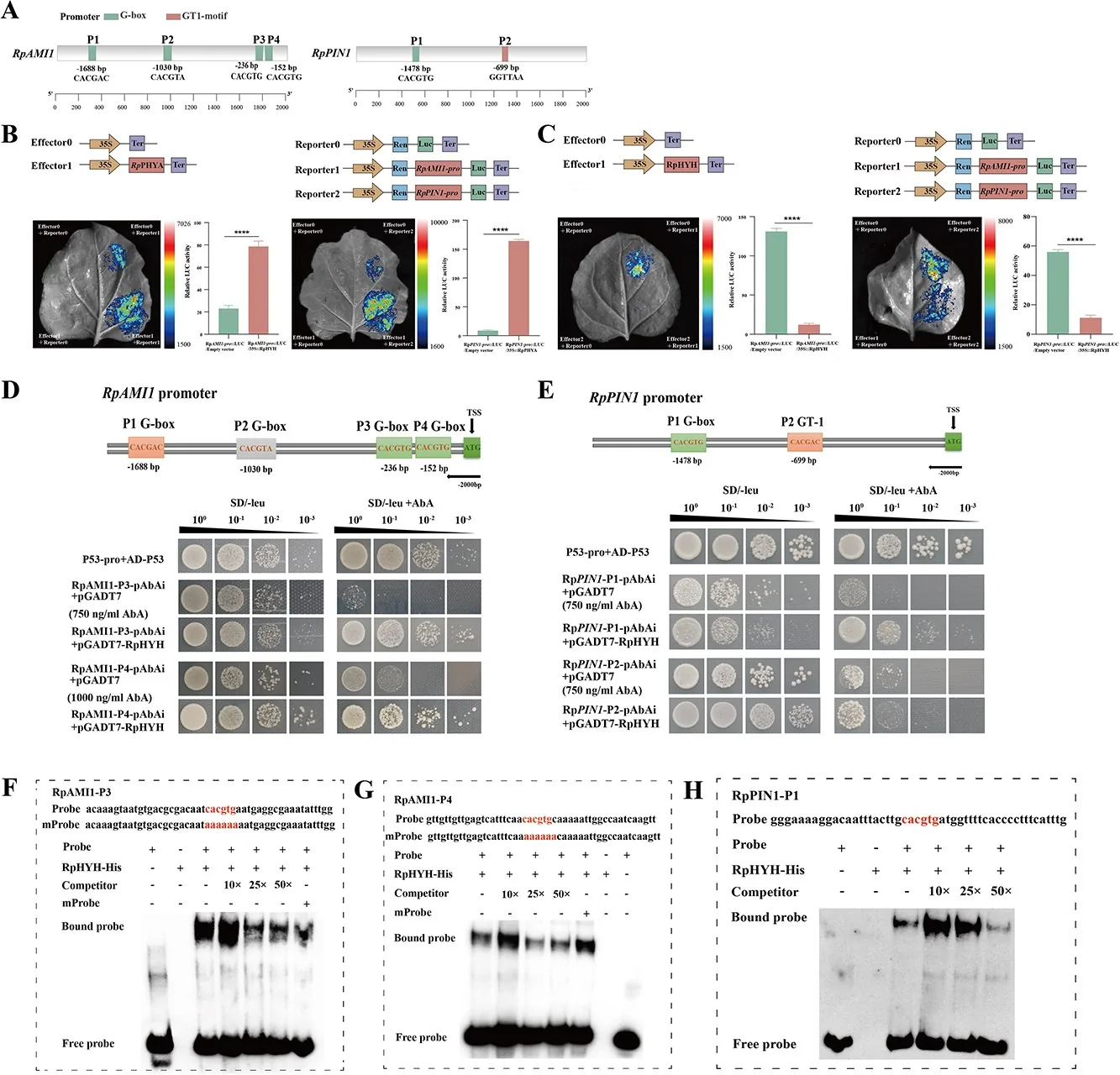
RpPHYA-associated module and RpHYH differentially regulate *RpAMI1* and *RpPIN1* expression by binding to their promoters. (A) Prediction analysis of light-responsive *cis*-acting elements in the promoter regions of *RpAMI1* and *RpPIN1*. (B) LUC reporter assays showing that the RpPHYA-associated regulatory module activates their promoter activities. (C) LUC reporter assays showing that RpHYH represses the promoter activities of *RpAMI1* and *RpPIN1*. (D) Y1H analysis showing that RpHYH bind to the *RpAMI1* promoter. (E) Y1H analysis showing that RpHYH bind to the *RpPIN1* promoter. (F) EMSA showing that RpHYH binds to the P3 site of the *RpAMI1* promoter. (G) EMSA showing that RpHYH binds to the P4 site of the *RpAMI1* promoter. (H) EMSA showing that RpHYH binds to the P1 site of the *RpPIN1* promoter. Statistical significance is indicated as follows: *P* < 0.05, *P* < 0.01, *P* < 0.001, *P* < 0.0001.

Furthermore, within the WGCNA Meturquoise module, which was negatively correlated with ELL14 (*r* = −0.98), RpHYH was identified. Phylogenetic analysis showed that RpHYH clusters with *Arabidopsis* HYH and is a paralog of HY5 (Fig. S9A), and subcellular localization indicated its presence in both the nucleus and cytoplasm (Fig. S9B). Previous studies have shown that AtHY5 represses AS regeneration in Arabidopsis by inhibiting *AtWUS* expression [9]. Consistently, *RpHYH* expression under CK conditions was significantly higher than under ELL14 (Fig. S7B). To investigate whether RpHYH regulates AS regeneration under normal light by affecting auxin biosynthesis and transport, LUC assays were performed, showing that RpHYH significantly repressed the expression of *RpAMI1* and *RpPIN1* (Fig. 4C). Y1H assays and EMSA further demonstrated that RpHYH binds to the P3 and P4 sites of the *RpAMI1* promoter (Fig. 4D, F, and  G) and to the P1 site of the *RpPIN1* promoter (Fig. 4E and H). Collectively, RpPHYA-associated regulatory module activates the expression of *RpAMI1* and *RpPIN1* under ELL14 treatment, promoting auxin biosynthesis and transport, whereas under CK, RpHYH represses the expression of *RpAMI1* and *RpPIN1*, thereby limiting AS regeneration in the leaves.

### ELL14 reduces CHG methylation to enhance AS regeneration

DNA methylation broadly participates in AS regeneration [27, 43]. In addition, within the set of DEGs, we identified several genes associated with DNA methylation (Fig. S10A). To further investigate the role of DNA methylation, 5-azacytidine (5-azaC) (50 μM), a DNA methylation inhibitor, was added to the AS induction medium under CK and ELL14 conditions (Fig. 5A–C). The number of shoots and regeneration frequency were measured at 30, 40, and 50 days after culture. The results showed that both the average number of AS and the regeneration frequency were significantly higher under the ELL14 treatment than under CK. However, treatment with 5-azaC largely reduced these differences at 30, 40, and 50 days (Fig. 5A–C), indicating that DNA methylation is involved in the regulation of ELL14-mediated AS regeneration. Subsequently, whole-genome bisulfite sequencing sequencing (WGBS) was performed on leaf explants collected at 8, 11, and 14 days under CK and ELL14 treatments. The results indicated that ELL14 treatment reduced CHG methylation (Fig. 5D) at all three time points and CHH methylation at 14 days (Fig. S10B) whereas CG methylation remained largely unchanged (Fig. S10C). Differentially methylated regions (DMRs) were quantified for the three time points (Fig. 5E–G). During AS regeneration, the number of downregulated CHG DMRs gradually increased, while upregulated CHG DMRs decreased (Fig. 5E–G). The number of differentially methylated cytosines (DMCs) was also quantified (Fig. S10D and E), showing that under ELL14, upregulated CHG DMCs gradually decreased (Fig. S10D), whereas downregulated DMCs in all three types increased over time (Fig. S10E). Hierarchical clustering of DMR methylation levels further revealed that CHG methylation was markedly reduced at 14 days under ELL14 (Fig. S10F). We then integrated the DMRs with the corresponding DEGs at 14 days (Fig. S10G and H), finding that genes upregulated under ELL14 exhibited lower CHG methylation levels compared to CK. This suggests that ELL14 may activate AS regeneration-related genes by reducing CHG methylation levels, thereby contributing to enhanced AS regeneration.

**Figure 5:**
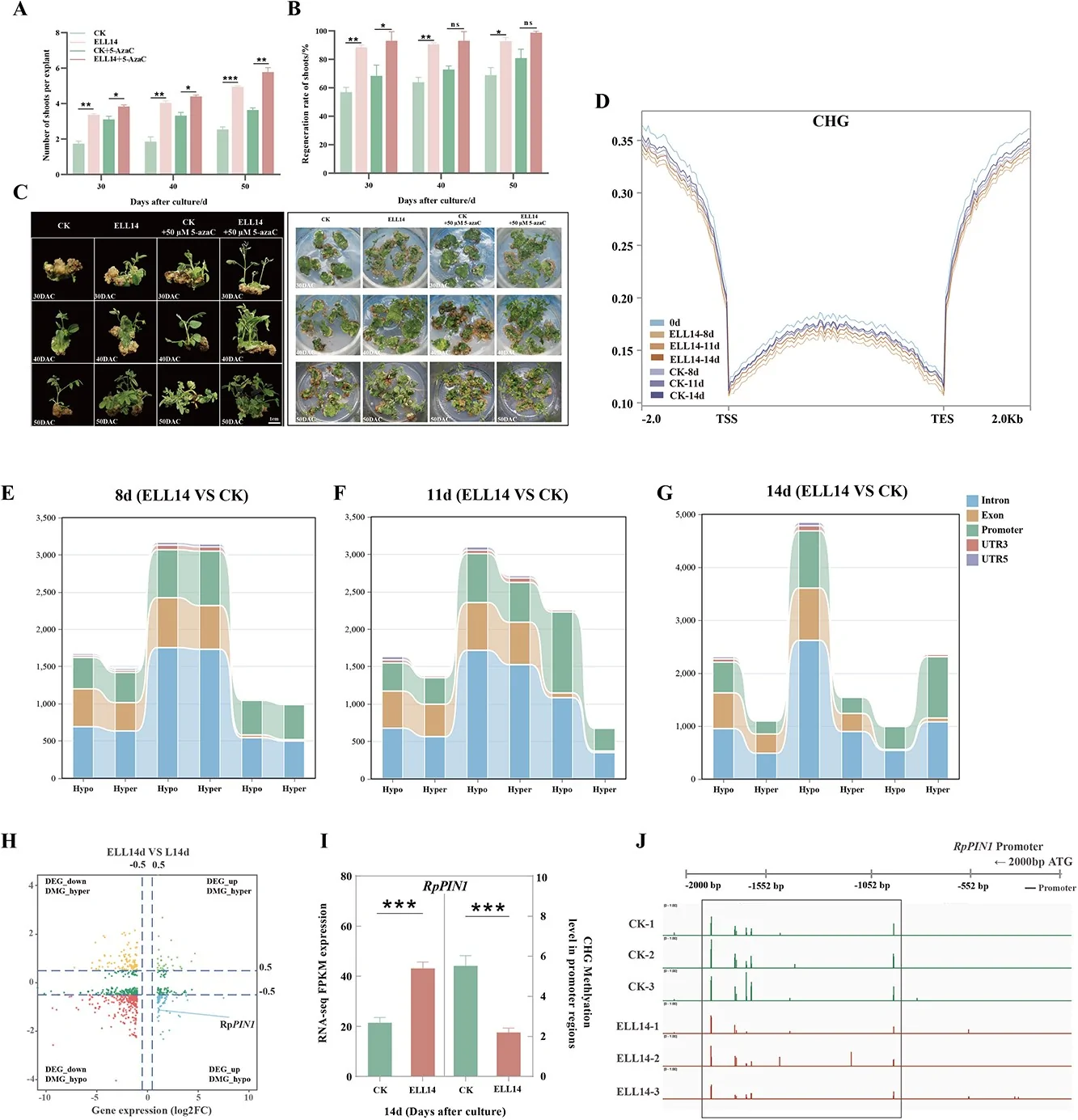
Effects of the DNA methylation inhibitor 5-azaC on AS regeneration and integrative analysis of genome-wide methylation remodeling and its association with DMRs, DEGs, and RpPIN1 regulation under CK and ELL14. (A and B) Quantification of AS number and regeneration efficiency in *R. pseudoacacia* leaf explants at 30, 40, and 50 days after inoculation following treatment with the DNA methylation inhibitor 5-azaC under CK and ELL14. (C) Phenotypic observation of AS regeneration from *R. pseudoacacia* leaf explants under CK and ELL14 with 5-azaC. (D) Genome-wide DNA methylation levels in CHG types under CK and ELL14. (E–G) Numbers of hypermethylated and hypomethylated DMRs at 8, 11, and 14 days under CK and ELL14. (H) Integrated analysis of DEGs and DMRs at 14 days under CK and ELL14. (I) The expression level and DNA methylation status of *RpPIN1* at 14 days. (J) Visualization of DNA methylation levels across the *RpPIN1* promoter under CK and ELL14 at 14 days.

### ELL14 promotes Rp*PIN1* expression by reducing CHG methylation in its promoter

To investigate which genes are regulated by DNA methylation during ELL14-enhanced AS regeneration, we quantified and visualized the numbers of DEGs and differentially methylated genes (DMGs) across three time points (Fig. S10I). Four-quadrant analysis revealed that a substantial proportion of DEGs were accompanied by changes in DNA methylation (Fig. 5H). Consistent with our previous results showing that *RpPIN1* is transcriptionally activated under ELL14, *RpPIN1* was among the genes exhibiting increased expression and reduced DNA methylation under ELL14 at 14 days (Fig. 5I). The methylation levels of the *RpPIN1* promoter were visualized under CK and ELL14, revealing that the CHG methylation in the −1900 to −800 bp region was significantly lower under ELL14 than CK (Fig. 5J). We also examined *RpPIN1* expression after 5-azaC treatment under both CK and ELL14. The results showed that *RpPIN1* was significantly upregulated under both treatments (Fig. 6A). This further indicates that reduced DNA methylation in the *RpPIN1* promoter enhances *RpPIN1* expression. To identify the specific methylated regions and sites within the *RpPIN1* promoter, 5-azaC treatment combined with GUS staining and McrBC-PCR were performed (Fig. 6B and C). We divided the promoter into three regions: P1, P2, and P3, containing all 9-, 8-, and 1-CHG site, respectively (Fig. 6B). GUS assays showed that 5-azaC application markedly enhanced GUS activity in the P1 and P2 regions, whereas no obvious change was observed in the P0 and P3 region (Fig. 6B). Similarly, for McrBC-PCR analysis, the P2 fragment was shortened and named p2 (−1880 to −1509 bp, still containing eight CHG sites), and the P3 fragment was shortened as p3 (−955 to −653 bp, still containing one CHG site). Compared to the negative and positive controls (Table S6), both p2 and p3 were methylated under CK and ELL14. Methylation at the p2 site was significantly lower, whereas no significant difference was observed at p3 (Fig. 6C). These results indicate that p2 is a region susceptible to DNA methylation. Bisulfite sequencing PCR (BSP-PCR) was conducted to further investigate the specific differentially methylated sites. The results showed that all eight CHG sites in the p2 region exhibited significant differences between CK and ELL14 (Fig. 6D; Table S7), with CHG methylation levels markedly higher under CK than ELL14 (Fig. 6E). These results indicate that, in addition to transcriptionally activating *RpPIN1* expression via the RpPHYA-associated module, ELL14 also promotes *RpPIN1* expression by reducing CHG-type DNA methylation in its promoter.

**Figure 6:**
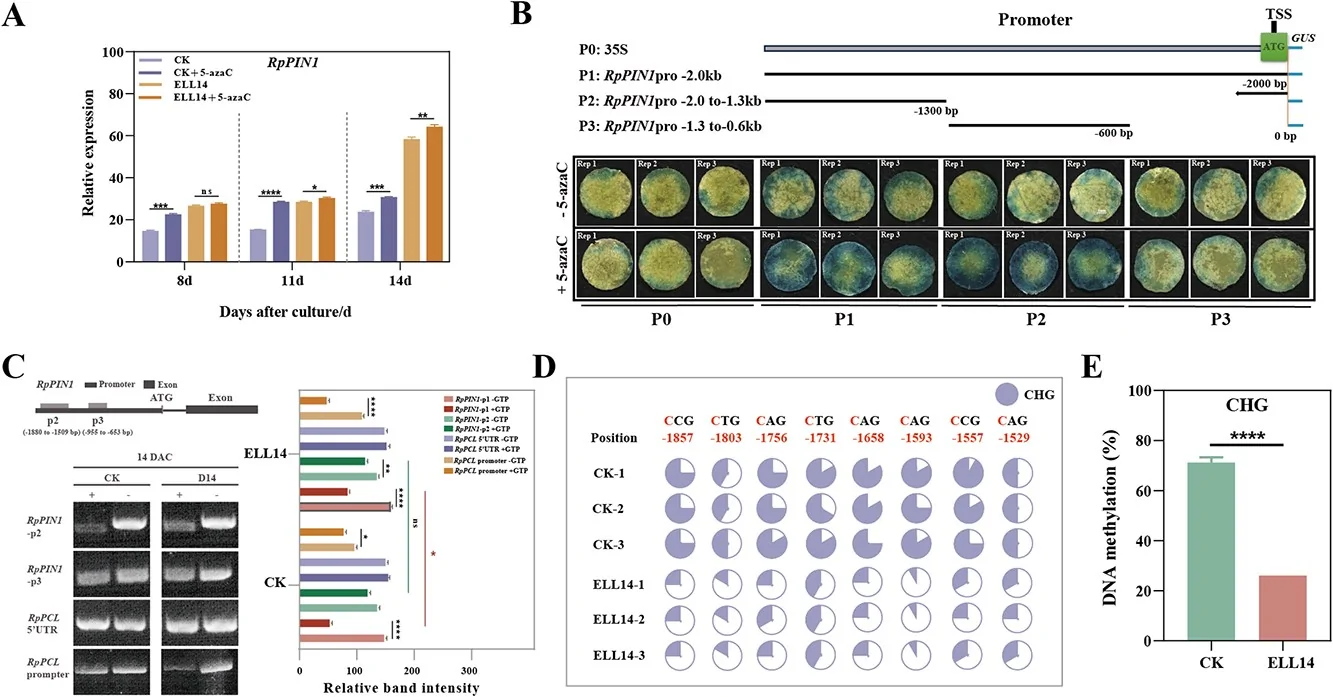
Dynamic CHG methylation of the RpPIN1 promoter regulates its expression during AS regeneration under CK and ELL14. (A) Changes in *RpPIN1* expression following treatment with the DNA methylation inhibitor 5-azaC under CK and ELL14. (B) Segmental promoter-GUS staining assay for validation of DNA methylation within the *RpPIN1* promoter region. TSS, transcription start site. (C) McrBC-PCR analysis validating methylated regions within the *RpPIN1* promoter. (D) BSP-PCR validation of specific DNA methylation sites within the *RpPIN1* promoter. (E) Quantification of CHG methylation levels at the *RpPIN1* promoter in leaf explants under CK and ELL14 at 14 days. Statistical significance is indicated as follows: *P* < 0.05, *P* < 0.01, *P* < 0.001, *P* < 0.0001.

### RpPHYA-associated module and RpHYH coordinately regulate RpROS1-mediated CHG demethylation to modulate *RpPIN1* expression

Given that reduced CHG methylation promotes *RpPIN1* expression and AS regeneration, we further investigated the genes encoding enzymes that regulate CHG methylation. Among them, the CHG-specific DNA demethylase *RpROS1* [29] exhibited a modest increase in transcript levels under ELL14 compared to CK (Fig. S11A). To determine whether the reduction in *RpPIN1* promoter methylation under ELL14 is mediated by RpROS1-dependent CHG demethylation, we performed LUC assays and genetic transformation in *R. pseudoacacia* calli (Fig. 7A and B). Overexpression of *RpROS1* significantly enhanced LUC activity driven by the *RpPIN1* promoter (Fig. 7A). In addition, *RpPIN1* expression was markedly increased in RpROS1-overexpressing calli compared to the wild type (Fig. 7B and S11B). These results demonstrate that RpROS1 promotes *RpPIN1* expression. To verify whether this upregulation involves promoter demethylation, BSP-PCR analysis was performed on genomic DNA from wild-type (WT) and *RpROS1*-overexpressing calli. The results showed that CHG methylation of the *RpPIN1* promoter was significantly reduced in the overexpressing lines (Fig. 7C and D; Table S8), suggesting that RpROS1 enhances *RpPIN1* expression by reducing promoter CHG methylation.

**Figure 7:**
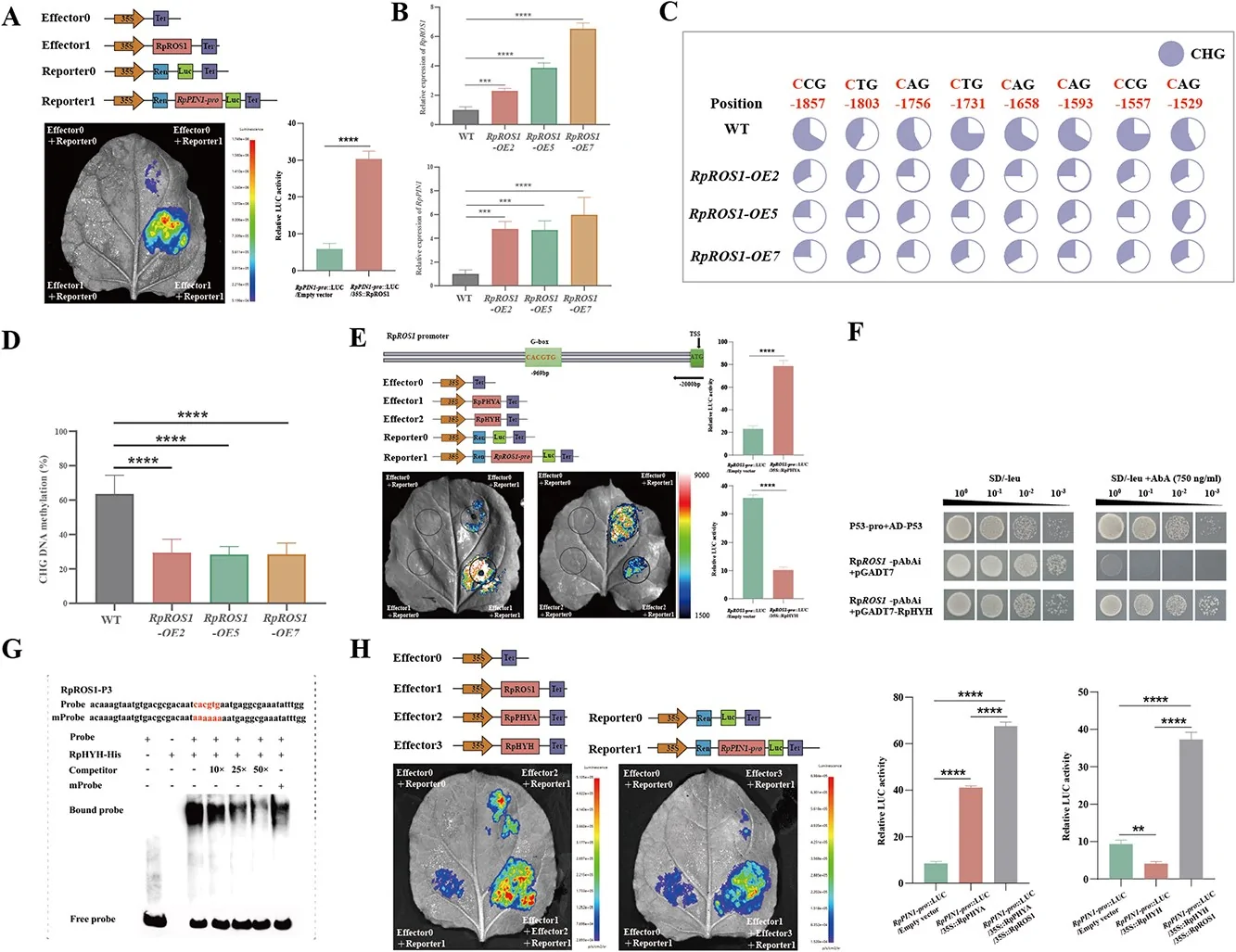
Regulation of RpPIN1 by the CHG-type DNA demethylase RpROS1, and upstream control of *RpROS1* by the RpPHYA-associated module and RpHYH during AS regeneration in *R. pseudoacacia*. (A) LUC assays examining the effect of Rp*ROS1* overexpression on *RpPIN1* expression. (B) Expression levels of *RpROS1* and *RpPIN1* following *RpROS1* overexpression in callus tissue of *R. pseudoacacia*. (C) BSP-PCR analysis of specific CHG methylation sites within the *RpPIN1* promoter in WT and *RpROS1*-OE calli. (D) Quantification of CHG methylation levels at the *RpPIN1* promoter in WT and *RpROS1*-overexpressing calli. (E) LUC assays examining the effects of RpPHYA-associated regulatory module and RpHYH on the promoter activity of *RpROS1*. (F) Y1H assays validating the binding of RpHYH to the *RpROS1* promoter. (G) EMSA showing the direct binding of RpHYH to the *RpROS1* promoter. (H) LUC assays assessing the effects of *RpROS1* overexpression on *RpPIN1* expression in the presence of RpPHYA-associated module or RpHYH-mediated regulation of the *RpPIN1* promoter, together with DNA methylation modification. Statistical significance is indicated as follows: *P* < 0.05, *P* < 0.01, *P* < 0.001, *P* < 0.0001.

Given that *RpROS1* showed a modest increase in expression under ELL14, we further examined whether it is regulated by ELL14. LUC assays showed that the RpPHYA-associated module activates *RpROS1* expression (Fig. 7E). Promoter analysis revealed a G-box element (Fig. 7E). To further assess the role of the G-box element in this regulatory process, we performed mutation analysis of the G-box in the *RpROS1* promoter. The results showed that mutation of the G-box abolished the ability of RpPHYA-associated module to activate *RpROS1* promoter activity (Fig. S11C), indicating that the transcriptional activation of *RpROS1* by the RpPHYA-associated module depends on the G-box *cis*-element. We further analyzed the interaction between RpPHYA and the G-box using Y1H and EMSA assays (Fig. S11D and E). The Y1H results showed that RpPHYA could weakly activate G-box-containing reporter genes in yeast (Fig. S11D), whereas EMSA results indicated that RpPHYA alone could not stably bind to the G-box sequence *in vitro* (Fig. S11E). These results suggest that RpPHYA may not directly bind to DNA in a stable manner, but more likely participates in G-box-mediated transcriptional regulation through interactions with other factors, which may, at least in part.

Additionally, LUC, Y1H, and EMSA assays demonstrated that RpHYH suppresses *RpROS1* expression (Fig. 7E–G). Further LUC analyses investigated the interplay between RpPHYA/RpHYH-mediated transcriptional regulation and RpROS1-mediated CHG demethylation of the *RpPIN1* promoter (Fig. 7H). The results indicated that RpROS1 and RpPHYA-associated module act synergistically to promote *RpPIN1* expression (Fig. 7H). Consistently, treatment with 5-azaC increased *RpPIN1* expression under both CK and ELL14 but did not fully abolish the difference between the two treatments (Fig. 6A), indicating that *RpPIN1* is regulated both by RpPHYA-mediated transcriptional activation and RpROS1-mediated demethylation. In addition, RpROS1 and RpHYH exert antagonistic effects, with the positive influence of RpROS1 being predominant (Fig. 7H).

### *RpPHYA*, *RpAMI1*, *RpPIN1*, and *RpROS1* promote AS regeneration in *Arabidopsis*

To further assess the roles of these key genes in AS regeneration, *RpPHYA*, *RpAMI1*, *RpPIN1*, and *RpROS1* were individually cloned into the pBI121 overexpressing-vector and transformed into *Arabidopsis* (Fig. S11F and H–J). WT and transgenic lines were first cultured on callus-induced medium (CIM) for 8 days to induce callus. As shown in Fig. 8A, the overexpression (OE) lines produced noticeably more callus than WT. Subsequently, AS regeneration was evaluated on shoot-induced medium (SIM) after 15 days. Approximately 6.9 ± 0.88 shoots per 1.5 cm were regenerated from WT root explants, whereas 13.7 ± 1.25, 14.9 ± 1.63, 15.2 ± 1.55, and 11.0 ± 1.32 shoots per 1.5 cm were regenerated from *RpPHYA-OE*, *RpAMI1-OE*, *RpPIN1-OE*, and *RpROS1-OE* lines, respectively. (Fig. 8B–G), indicating that these genes positively contribute to AS regeneration. Root explants of *RpHYH-OE*, *phya*, *ami1*, and *pin1* mutants were analyzed alongside OE lines (Fig. S11G and S12). They regenerated an average of 4.6 ± 0.84, 1.9 ± 0.74, 4.0 ± 0.63, and 2.8 ± 0.79 shoots per 1.5 cm, respectively, significantly lower than WT, indicating reduced AS regeneration in these mutants (Fig. S12A–C). Based on these findings, a model for ELL14-mediated AS regeneration in *R. pseudoacacia* leaves is proposed (Fig. 8H). Under ELL14, the RpPHYA-associated regulatory module promotes auxin biosynthesis and transport by transcriptionally activating *RpAMI1* and *RpPIN1*, thereby enhancing AS regeneration. The RpPHYA module also upregulates *RpROS1*, leading to CHG demethylation at the *RpPIN1* promoter and further enhancing its expression. In contrast, under normal light conditions, RpHYH restricts the regenerative potential of leaf explants by repressing *RpAMI1* and *RpPIN1* expression, thereby limiting auxin biosynthesis and transport and constraining AS formation. RpHYH also modulates *RpROS1* expression to maintain CHG methylation at the *RpPIN1* promoter, further restricting *RpPIN1* expression.

**Figure 8:**
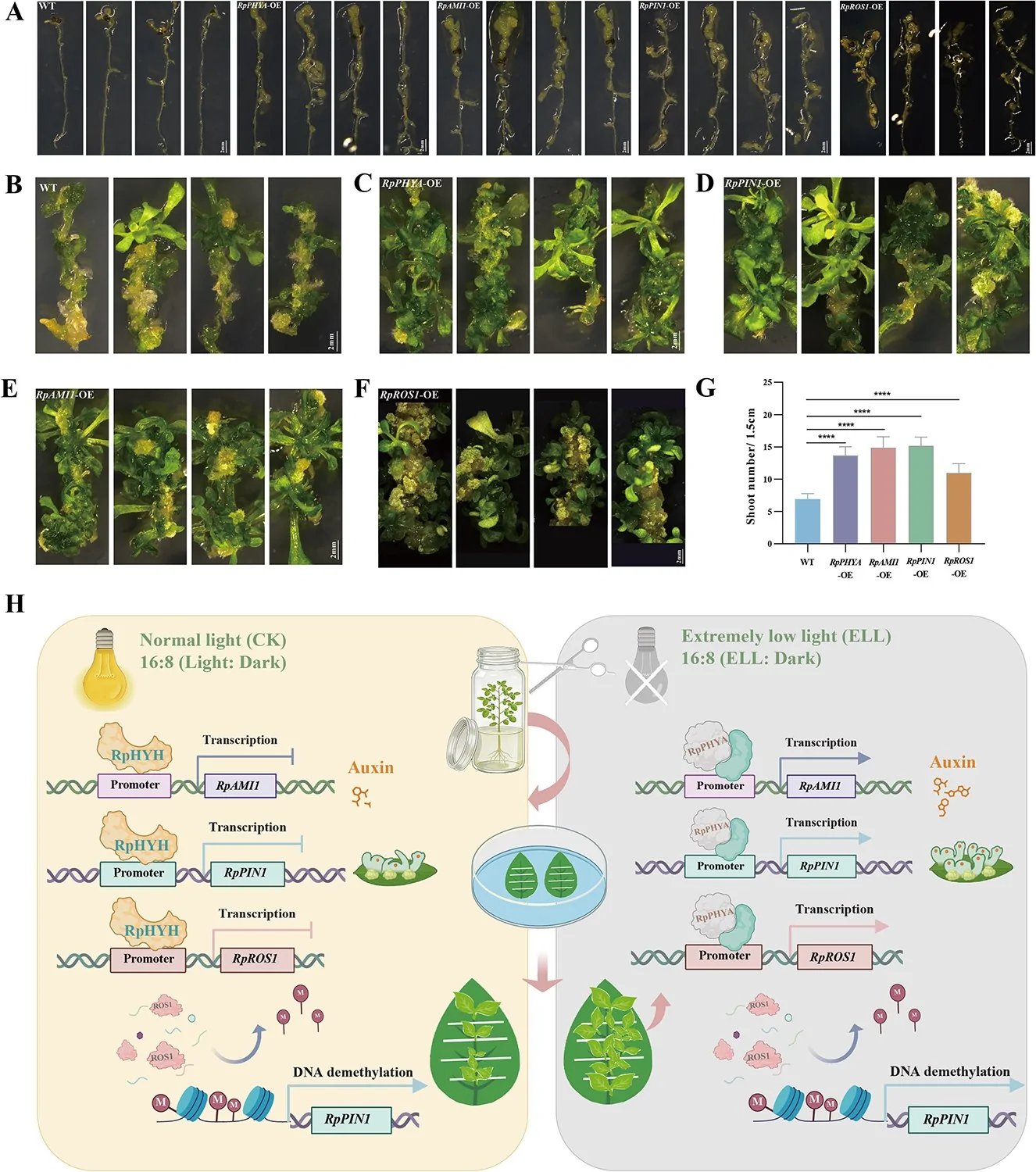
Functional validation of *RpPHYA*-, *RpAMI1*-, *RpPIN1*-, and *RpROS1*-mediated regulation of AS regeneration and a proposed model under CK and ELL14. (A) Phenotypic observation of callus differentiation in *Arabidopsis* WT, *RpPHYA*-OE, *RpAMI1*-OE, *RpPIN1*-OE, and *RpROS1*-OE lines. (B–F) Phenotypic observation of AS regeneration from root explants of the same *Arabidopsis* lines, shown in panels B–F. (G) Quantification of AS numbers regenerated from root explants of the indicated *Arabidopsis* lines. (H) Schematic model illustrating the promotive effect of ELL14 on AS regeneration from *R. pseudoacacia* leaf explants. Statistical significance is indicated as follows: *P* < 0.05, *P* < 0.01, *P* < 0.001, *P* < 0.0001.

## Discussion

### ELL14 shape AS regeneration efficiency and competence

Light conditions exert complex regulatory effects on AS regeneration, and plant responses to light conditions vary among species, genotypes, and explants [4, 8, 9, 35, 44]. In woody plants, ELL14 has been widely applied as a practical strategy to enhance AS regeneration and facilitate genetic transformation. For instance, a period of light-shading (low light) increased the number of shoots regenerated from stem explants of sweet orange and grapefruit by approximately nine and five shoots, respectively (Marutani-Hert et al., 2012). However, the response to low light is not universal and is often explant-dependent. For instance, Liu and Bao demonstrated that light-shading promoted AS regeneration from *Platanus* hypocotyls but inhibited regeneration from leaf explants [6]. AS regeneration in *R. pseudoacacia* is constrained by strong genotype dependence and frequent tissue browning [37]. In this study, we used detached leaves of *R. pseudoacacia* as explants to systematically investigate the effects of ELL on AS regeneration. Our results showed that ELL14 significantly promoted AS regeneration from leaves. Morphological and histological observations indicated that ELL14 accelerated the initiation and formation of shoot primordia. Further RNA-seq and gas chromatography–mass spectrometry (GC–MS) analyses revealed that ELL14 enhanced AS regeneration by promoting auxin biosynthesis and polar transport. Additionally, ELL14 reduced CHG methylation levels at the *RpPIN1* promoter, suggesting that auxin transport processes are also regulated by DNA methylation. This study provides the first systematic evidence that ELL14 promotes AS regeneration in *R. pseudoacacia* through the coordinated regulation of auxin-related transcription and DNA demethylation, thereby offering new mechanistic insights into environmentally controlled shoot regeneration in woody plants.

### RpPHYA participates in the regulation of AS regeneration in excised *R. pseudoacacia* leaves under ELL14

PHYA is a key photoreceptor that senses low-light environments [45–47]. Early studies proposed that phytochromes could participate in transcriptional regulation at the promoter level. PHYB forms a complex with the transcription factor PIF3 *in vitro* and binds to DNA, thereby proposing a conceptual model in which phytochromes regulate gene expression at the promoter level [48]. Subsequently, direct evidence showed that PHYA is recruited to the promoter of the anthocyanin biosynthetic gene *CHALCONE SYNTHASE* and cooperatively activates its transcription, thereby establishing the role of PHYA in nuclear transcriptional regulation [49]. Notably, PHYA lacks a canonical DNA-binding domain. Therefore, its transcriptional regulatory function in the nucleus depends on PHYA-containing complexes composed of transcription factors and co-regulatory proteins [42].

ChIP-seq analyses have shown that PHYA-associated complexes are frequently enriched at classical light-responsive *cis*-elements such as the G-box [42] and often act synergistically with light-responsive transcription factors such as HY5 to convert light signals into transcriptional outputs. In this study, our Y1H assays revealed that RpPHYA could weakly activate reporter gene expression driven by the promoters of *RpAMI1*, *RpPIN1*, and *RpROS1*. Given that PHYA is typically characterized as a non-DNA-binding protein, this transactivation in a heterologous system most likely results from the ‘bridging’ effect of endogenous yeast transcription factors. Previous studies have established that yeast bZIP proteins, such as Sko1p and the AP-1-like factor Yap1p, can specifically recognize and bind to G-box and ACGT-core elements commonly found in plant promoters [50, 51]. Although these endogenous yeast transcription factors may differ from the native interaction partners in *R. pseudoacacia*, together with the EMSA results showing that RpPHYA alone cannot stably bind to target promoters, these data to some extent support a model in which RpPHYA associates with target promoters indirectly through protein–protein interactions rather than by directly binding to DNA.

These findings suggest that RpPHYA likely integrates low light signals in detached *R. pseudoacacia* leaves through a conserved complex-mediated mechanism, thereby reprogramming transcriptional networks associated with AS regeneration. In this context, although we identified an RpPHYA-associated regulatory module and its downstream targets, the transcription factors that directly recognize G-box elements and assemble functional complexes with RpPHYA remain to be identified, representing an important direction for future research. Previous studies have investigated the formation of transcriptional complexes involving PHYA. FAR-RED ELONGATED HYPOCOTYL 1 (FHY1) has been proposed as a key co-regulatory factor linking PHYA to certain transcription factors; however, PHYA-mediated promoter binding and transcriptional regulation are not entirely dependent on the FHY1 pathway. For instance, PHYA binding to the *NAC019* promoter occurs independently of FHY1, and loss of FHY1 does not completely abolish PHYA signaling output, indicating that additional auxiliary factors are involved in mediating PHYA nuclear functions [42, 52]. In our study, although we identified an RpPHYA-associated regulatory module and its downstream targets, the transcription factors that directly recognize G-box elements and assemble functional complexes with RpPHYA remain to be identified, representing an important direction for future research.

### Crosstalk between ELL signaling and auxin pathways during AS regeneration

Previous studies have demonstrated a close functional crosstalk between light conditions and auxin signaling [26, 53]. During organ regeneration, ELL profoundly influence regeneration efficiency and spatial patterning by modulating auxin biosynthesis, polar transport, and downstream signaling pathways [22, 44]. Previous studies have demonstrated that PHYA modulates auxin signaling by affecting the stability and activity of key components in the auxin signaling pathway. For example, in *Arabidopsis*, PHYA responses by interfering with the TIR1/AFBs-AUX/IAA module [54]. In the present study, transcriptomic analyses revealed that DEGs under ELL were significantly enriched in pathways related to auxin biosynthesis and polar auxin transport, further supporting a central role of auxin in mediating ELL-induced AS regeneration in *R. pseudoacacia*.

AMI1 encodes a key enzyme in the IAM pathway that converts IAM into IAA. Although the IAM pathway has long been considered a minor route of auxin biosynthesis, recent studies have shown that it can substantially contribute to local auxin accumulation during specific developmental stages and environmental responses in crops such as wheat and rice [55]. In this study, *RpAMI1* expression was significantly upregulated under ELL, and application of the auxin biosynthesis inhibitor yuc markedly attenuated the promotive effect of ELL on AS regeneration. These results indicate that the IAM pathway plays an underestimated yet important role in AS regeneration in woody plants and further validates auxin as a key downstream mediator of ELL.

Auxin polar transport represents a critical regulatory layer determining the spatial pattern of organogenesis [56]. PIN1-mediated directional auxin efflux is essential for the establishment of local auxin maxima, a process that is indispensable for shoot primordium initiation and maintenance [26, 53]. Our results show that ELL14 significantly enhances *RpPIN1* expression, concomitant with a pronounced acceleration of shoot primordium formation. This suggests that ELL14 promotes AS regeneration by reinforcing polar auxin transport and facilitating the establishment of functional auxin gradients within explants. Furthermore, genetic functional analyses in *Arabidopsis* demonstrated that overexpression of *RpAMI1* or *RpPIN1* resulted in the formation of a greater number of shoots compared to wild-type plants, providing additional evidence for the conserved roles of auxin biosynthesis and polar transport during AS regeneration. Collectively, from the perspective of ELL14-auxin crosstalk, this study establishes a regulatory module by which ELL14 promotes AS regeneration in woody plants, offering a refined mechanistic framework for understanding how ELL14 fine-tune regenerative capacity through hormonal pathways.

### Crosstalk between light signaling and DNA methylation dynamics during AS regeneration

DNA methylation plays a significant role in plant growth and development [57], and recent studies have further highlighted its significance in AS regeneration [11, 12, 27]. Maintenance and dynamic regulation of DNA methylation at CG, CHG, and CHH contexts can profoundly influence gene expression, thereby modulating cell fate reprogramming and the initiation of shoot primordia. For instance, in *Arabidopsis*, *met1* mutants exhibit upregulation of cytokinin-related genes such as *ARR1* and *ARR10*, promoting AS regeneration [5]. Moreover, non-CG methylation has also been shown to contribute to AS regeneration, as observed in *drm1 drm2 cmt3* (*ddc*) triple mutants, which relieve the transcriptional repression of WUS, allowing root explants to form AS directly on SIM without passing through CIM stage [28].

DNA methylation as an important interface linking ELL14 to transcriptional regulation. For instance, in strawberry, bagging treatment delays fruit maturation, and the blue-light receptor CRY1 regulates DNA methylation via ARGONAUTE 4 at the promoter of *FaCYP707A4*, repressing its expression and promoting ABA accumulation to accelerate fruit ripening [58]. Building upon this knowledge, we analyzed DNA methylation dynamics in *R. pseudoacacia* leaf explants under ELL14 and CK. WGBS revealed that ELL14 broadly reduced CHG methylation levels across the genome. Mechanistically, the RpPHYA-associated regulatory module directly activated the CHG-specific DNA demethylase RpROS1, thereby promoting CHG demethylation. In contrast, under normal light conditions, higher CHG methylation levels were maintained, likely due to the partial repression of *RpROS1* by RpHYH. Functional analysis further demonstrated that overexpression of *RpROS1* significantly enhanced AS formation compared to wild-type plants, providing the first direct evidence that *RpROS1* positively regulates AS regeneration. Collectively, our results establish an ELL-DNA demethylation-auxin regulatory axis in which RpPHYA-mediated activation of *RpROS1* reshapes DNA methylation landscapes to fine-tune auxin transport during regeneration. This study provides the first evidence that ELL-dependent DNA methylation dynamics are functionally integrated into the regulation of AS in woody plants.

### Dual transcriptional and DNA methylation balances AS regeneration and developmental stability

Plant developmental regulation often does not rely on a single transcriptional event but is achieved through the coordinated action of multiple regulatory layers to ensure stable expression of key genes in specific spatial and temporal contexts. Accumulating evidence indicates that such dual regulatory mechanisms represent a common strategy for integrating environmental cues with endogenous developmental programs. For instance, during flowering regulation in *Arabidopsis*, the key flowering repressor FLOWERING LOCUS C is not only controlled by transcription factor complexes but is also regulated via epigenetic modifications, including H3K27me3 and DNA methylation at its promoter and gene body, enabling stable control of flowering timing [59, 60]. In the present study, ELL14 was found to regulate AS regeneration in *R. pseudoacacia* through a dual mechanism. On one hand, the RpPHYA-associated complex activates the transcription of *RpPIN1*, promoting auxin polar transport, thereby establishing functional auxin gradients. On the other hand, the RpPHYA complex upregulates the DNA demethylase *RpROS1*, which reduces CHG methylation at the *RpPIN1* promoter, further enhancing its transcription and reinforcing auxin gradient formation and shoot primordium positioning. This transcriptional and epigenetic dual regulation enables fine-tuned spatiotemporal expression of key auxin transport genes, supporting both the initiation and maintenance of shoot primordia. Furthermore, under normal light, HYH represses auxin biosynthesis and transport, limiting AS regeneration, while higher DNA methylation may impose an “environmental constraint” that prevents excessive primordium formation and maintains developmental stability. These features highlight how plants convert environmental signals into stable developmental decisions and provide key insights into the regulatory strategies underlying regeneration under complex environmental conditions.

## Conclusion

This study provides novel mechanistic insights into how ELL14 enhances AS regeneration in *R. pseudoacacia* leaf explants. We show that RpPHYA acts as a central integrator of ELL, promoting auxin biosynthesis and polar transport via a regulatory module that activates *RpAMI1* and *RpPIN1* transcription. Concurrently, RpPHYA enhances *RpROS1*-mediated DNA demethylation at the *RpPIN1* promoter, further facilitating auxin transport and AS regeneration. In contrast, under normal light, RpHYH restricts auxin biosynthesis and transport, thereby limiting the regenerative potential of leaf explants. Together, these results demonstrate that ELL14 promotes AS regeneration through a dual regulatory mechanism-coordinating transcriptional activation via the RpPHYA-associated module and DNA methylation control of auxin transport genes-linking ELL signals to increased organogenic competence and providing a conceptual framework for improving clonal propagation in recalcitrant woody species.

## Materials and methods

### Materials and culture conditions

Diploid (‘PU18-22’, ‘P28-15’, ‘M28-34’) and tetraploid (‘38’, ‘A4’) plantlets of *R. pseudoacacia* maintained *in vitro* were used as experimental materials. All plantlets were cultured under controlled conditions (23 ± 2°C with a 16 h light/8 h dark photoperiod). Fully expanded leaves were excised from plantlets subcultured for 35–40 days, and 3–5 small wounds were gently made on the abaxial surface using a sterile scalpel. The wounded leaves were then placed on AS induction medium consisting of MS salts supplemented with 30 g·l^−1^ sucrose, 3 mg·l^−1^ 6-BA, 1·mg·l^−1^ KT, and 5.5 g·l^−1^ agar. Each treatment included 5–10 leaf explants per replicate, with six independent biological replicates.

Explants were exposed to four light treatments under a 16 h light/8 h dark photoperiod with identical dark periods. Light intensity during the light phase differed among treatments. Treatments included normal light (CK) and ELL for 7 (ELL7), 14 (ELL14), or 21 days (ELL21). After the treatment period, all explants were transferred to CK conditions. The average number of regenerated AS per explant was calculated as the total number of shoot primordia divided by the total number of inoculated explants. AS regeneration efficiency was defined as the percentage of explants producing at least one shoot primordium. Evaluations were performed at 30, 40, and 50 days after inoculation. The developmental morphology of AS was documented to assess the regenerative capacity among treatments.

### Radiance measurement

Leaf explants were subjected to ELL using a shading cloth. A field spectroradiometer was used to measure light irradiance (Fieldspec 4 HR NG). The fiber optic probe was vertically positioned toward the tissue culture room light for calibration. Measurements were taken at the same position under CK, and then repeated under ELL. Each measurement was performed in triplicate, and the mean irradiance under CK and ELL was calculated. Spectral radiance (W·m^−2^·sr^−1^·nm^−1^) was converted to spectral photon flux density (μmol·m^−2^·s^−1^·nm^−1^) assuming a Lambertian light field (*E* = π*L*). Photon flux density was calculated according to Planck’s equation and integrated over 400–700 nm to obtain total PPFD (Table S9).

### Morphological, histological, and anatomical analyses

Leaf explants that cultured under CK and ELL14 were observed under a stereomicroscope (M205FA, Leica) to record their morphological characteristics. Samples were subsequently fixed in a glutaraldehyde-paraformaldehyde solution for semi-thin sectioning (EM UC7, Leica), in FAA fixative (formalin-acetic acid-alcohol) for SEM (S-3400N, Hitachi), and in 4% paraformaldehyde fixative for *in situ* hybridization. Tissues embedded in resin were sectioned using an ultramicrotome and stained with 0.5% toluidine blue O for histological observation. For *in situ* hybridization, fixed samples were sectioned and hybridized with the *RpWUS* probe to determine the spatial expression pattern of *RpWUS* within leaf explants.

### DNA and RNA extraction, cDNA synthesis, and RT-qPCR

The leaf explants cultured at 8, 11 and 14 days under CK and ELL14 were collected and immediately frozen in liquid nitrogen upon collection and stored at −80°C for DNA and RNA extraction. Genomic DNA (DC104-01, Vazyme) and total RNA (RC411-01, Vazyme) were extracted, and first-strand cDNA (R312-01, Vazyme) was synthesized. Gene-specific primers were designed using Primer 5 software (primer sequence are listed in Table S10). Quantitative real-time PCR (RT-qPCR) was performed using Taq Pro Universal SYBR qPCR Master Mix (Q712-02, Vazyme) according to the manufacturer’s instructions. Each experiment was carried out with three biological replicates. All data were presented as mean ± standard deviation (SD) with *n* = 3. The relative expression levels of target genes were calculated using the 2^^(−ΔΔCT)^ method, and statistical analysis was performed by two-way analysis of variance.

### RNA-seq and differential gene expression analysis

The leaf explants cultured at 8, 11 and 14 days under CK and ELL14 were collected for total RNA extracting. Each sample was performed with three biological replicates. RNA quality was assessed prior to library preparation. mRNA was enriched using oligo(dT) magnetic beads, and sequencing libraries were constructed following standard protocols. Qualified libraries were sequenced on the BGI high-throughput sequencing platform DNBSEQ. DEGs at each developmental stage were identified based on filtered sequencing data. Functional enrichment analysis of DEGs was performed using GO and KEGG pathways via TBtools. ClueGO analysis was conducted using Cytoscape. Volcano plots of DEGs were generated using Rstudio 4.3.3.

### Auxin quantification and treatment with auxin biosynthesis and transport inhibitors

The leaf explants cultured at 8, 11, and 14 days under CK and ELL14 were collected for auxin level measurement. Each sample was performed with three biological replicates. Each sample was ground in liquid nitrogen and processed prior to analysis. Auxin content was determined using ultra-performance liquid chromatography (UPLC ExionLC™ AD) coupled with tandem mass spectrometry (MS/MS, QTRAP® 6500+). Mass spectrometric data were subjected to qualitative and quantitative analyses. The auxin biosynthesis inhibitor YUCCA monooxygenase inhibitor (yucasin) was used at 10 μM, and the auxin transport inhibitor NPA was applied at 20 μM. After filter sterilization, both inhibitors were added to the AS induction medium, and their effects on AS regeneration from leaf explants were then assessed.

### Weighted gene co-expression network analysis and network visualization

A total of 4875 DEGs were identified by taking the union of DEGs at ELL14-8 days, ELL14-11 days, and ELL14-14 days relative to their corresponding checks (CK-8 days, CK-11 days, and CK-14 days). Genes with expression levels below 0.5 were filtered out. WGCNA was conducted to associate the remaining DEGs with auxin precursor Trp, IAA, and light conditions (ELL14, CK). Modules showing high correlation with auxin content and ELL was selected for further analysis. Hub genes were identified within each module, and gene pairs with a correlation coefficient (*r*) ≥ 0.8 and *P* < 0.05 were used to construct the co-expression network. The network was visualized using Cytoscape software.

### Promoter *cis*-element prediction and dual-luciferase assay

The 2000 bp upstream promoter sequences of *RpAMI1*, *RpPIN1*, and *RpROS1* were extracted from the genome for *cis*-element prediction. The 2000 bp upstream promoter sequences of *RpAMI1*, *RpPIN1*, and *RpROS1*, with G-box elements mutated from ‘CACGTG’ to ‘AAAAAA’, were synthesized and cloned into vectors by Tsingke Biotechnology (Beijing, China). Promoter sequences were cloned into the reporter vector pGreenII 0800-LUC, while RpPHYA and RpHYH were cloned into the effector vector (pGreen-62-SK, 35S::TF). For the methylation validation experiment, the coding sequences (CDS) of *RpROS1* was cloned into the pCAMBIA1300-cHA overexpression vector. Effector and reporter constructs were introduced into *Agrobacterium tumefaciens* GV3101 (psoup19) and mixed at a 1:1 ratio. Transient transformation of *Nicotiana benthamiana* leaves was performed via needle injection. Luciferase activity was measured 48–72 h post-infiltration using a luminescence imaging system. All experiments were performed with at least three independent biological replicates, showing good consistency across experiments, and representative results are presented.

### Yeast one-hybrid assay

For the Y1H assay, 300 bp fragments of the target gene promoters (*RpAMI1*, *RpPIN1*, and *RpROS1*), including the predicted binding sites, were cloned into the pAbAi vector and subsequently transformed into Y1HGold competent cells. The CDS of RpPHYA and RpHYH were cloned into the pGADT7 vector to generate effector constructs. After confirming the absence of autoactivation, yeast transformants were cultured on SD/−Leu + AbA plates at 30°C for 3–5 days to assess growth. All experiments were performed with at least three independent biological replicates, showing good consistency across experiments, and representative results are presented.

### Electrophoretic mobility shift assay

For the EMSA, 40–60 bp sequences encompassing the predicted binding sites in the promoters of target genes were synthesized as probes (Table S11). Biotin-labeled probes were used as labeled probes, while unlabeled probes served as competitors. Mutant probes were generated by replacing all nucleotides within the binding sites with adenine (A). CDS of the transcription factors were cloned into expression vectors with histidine tag (His) or maltose-binding protein (MBP) tags for recombinant protein expression, and the proteins were subsequently purified (Fig. S13). Purified proteins were incubated with the synthesized probes, and DNA-protein complexes were analyzed by gel electrophoresis to assess binding interactions. All experiments were performed with at least three independent biological replicates, showing good consistency across experiments, and representative results are presented.

### Whole-genome bisulfite sequencing analysis

Genomic DNA was extracted from the samples for analysis. DNA methylation libraries were prepared by treating DNA fragments with bisulfite, which converts unmethylated cytosines (C) to thymine (T), followed by library construction and sequencing. After sequencing, raw reads were filtered for quality and aligned to the reference genome. Genome-wide cytosine coverage and methylation levels were then assessed for downstream analyses. Each sample was performed with three biological replicates.

### 5-Azacytidine treatment

A 50-μM 5-azaC was added to the AS induction medium for leaf explants. AS regeneration was assessed by measuring the average number of shoots per leaf and AS regeneration efficiency. These assessments were conducted under four treatments, including CK, CK with 5-azaC, ELL14, and ELL14 with 5-azaC at 30, 40, and 50 days of culture. Leaf samples were collected at 0, 8, 11, and 14 days for each treatment, rapidly frozen in liquid nitrogen, and stored at −80°C. Each sample was performed with six biological replicates. Expression levels of *RpPIN1* were quantified using RT-qPCR.

### DNA methylation analysis

To investigate whether the promoter region of *RpPIN1* is regulated by DNA methylation, the 2000 bp upstream sequence of *RpPIN1* was divided into three fragments: P1 (−2000 to 0 bp), P2 (−2000 to −1300 bp), and P3 (−1300 to −600 bp). Each fragment was cloned into the pCAMBIA1391::*GUS* reporter vector, with the empty vector serving as a control, and transiently expressed in tobacco leaves. A 100-μM solution of 5-azaC was applied to the leaves every 6 h. After 72 h, leaf discs were collected using a puncher, and GUS staining was performed using GUS staining kit (BN20175, Biorigin) to visualize promoter activity.

### Methylated DNA-specific restriction endonuclease B and C (McrBC)-PCR analysis

To assess DNA methylation levels in the promoter region of target genes, a semi-quantitative McrBC-PCR approach was applied. Genomic DNA was extracted from samples collected at four time points under CK and ELL14, and purified. The DNA was digested with McrBC polymerase (1234A, Takara) at 37°C for 3 h, and the reaction was terminated. The 5′ UTR of pectinase-like (*RpPCL)*, a region known to lack DNA methylation, was used as a negative control, while the *RpPCL* promoter, containing methylated regions, served as a positive control. Digested DNA was subjected to PCR amplification of the target region using a KOD One™ PCR Master Mix polymerase (KMM-101, ToYOBO) to ensure specificity. PCR products were resolved on 1.5% agarose gels, and the intensity of the bands was measured using Image J. Each sample was performed using three biological replicates.

### Bisulfite sequencing PCR analysis

To further assess the *RpPIN1* promoter methylation sites, genomic DNA was treated with the bisulfite conversion kit (D0068M, Beyotime) to convert unmethylated cytosines (C) to thymines (T), while methylated cytosines remained unchanged. The bisulfite-converted DNA was purified and amplified using specific primers targeting the *RpPIN1* promoter region. PCR products were purified, cloned into a T-vector, and transformed into DH5α competent cells. From each sample, 12 positive clones were randomly selected and sequenced. The methylation status of multiple CpG sites was analyzed with CyMATE (https://cymate.org/webscript/cymate.py), and methylation maps were generated. Each experiment was independently repeated three times, and the results were used to quantitatively compare the methylation levels of the *RpPIN1* promoter under CK and ELL14. Each sample was performed with three biological replicates.

### Agrobacterium-mediated transformation of *R. pseudoacacia* callus

Approximately 30-day-old plantlets of *R. pseudoacacia* were used for callus induction. Stem segments were excised and cultured on callus-inducing medium (MS supplemented with 30 g·l^−1^ sucrose, 4 mg·l^−1^ 6-BA, 0.6 mg·l^−1^ NAA, 2 mg·l^−1^ 2,4-D, and 5.5 g·l^−1^ agar). After 20 days, visible callus formation was observed. The stem segments were subsequently transferred to callus proliferation medium (MS supplemented with 30 g·l^−1^ sucrose, 1 mg·l^−1^ 6-BA, 0.4 mg·l^−1^ NAA, 2 mg/l 2,4-D, and 5.5 g·l^−1^ agar). After 3–5 days, the callus was inoculated with an *A. tumefaciens* suspension. Following 3 days of co-cultivation, the explants were transferred to the callus proliferation medium containing 50 mg·l^−1^ kanamycin. After 20 days post-infection, normally proliferating callus were selected for molecular verification.

### Genetic transformation of *Arabidopsis*

For the generation of overexpression lines, *Arabidopsis* plants at the inflorescence emergence stage with numerous unopened flower buds were inverted and immersed in *A. tumefaciens* suspension (OD_600_ ≈ 0.6–0.8) for 1 minute with gentle agitation. The treated plants were incubated in darkness for 24 h and then returned to standard long-day conditions (16 h light/8 h dark, 22°C) until seed maturation. T₁ seeds were collected and selected on 1/2 MS medium containing 50 mg·l^−1^ kanamycin. DNA analyses were performed for molecular verification. T₂ and T₃ seeds were obtained to ensure stable transgene integration, with T₃ seeds being germinated on selective medium. Verified overexpression lines and wild-type plants were germinated on 1/2 MS medium. For AS regeneration experiments, roots from 10-day-old seedlings were excised and cultured on CIM for 7 days. Subsequently, root explants were transferred to SIM and cultured for 21 days. AS regeneration frequency and the number of shoots were measured and analyzed. The *Arabidopsis* T-DNA insertion mutant lines *phya* (SALK_014575C), *ami1* (SALK_019823C), and *pin1* (SALK_097144C) were obtained from Arashare (Shanghai, China).

### Data analysis

Data were organized and summarized using Microsoft Excel 2021. Statistical analyses and graphical representations were performed using IBM SPSS Statistics 26, GraphPad Prism 9, R Studio, and Adobe Illustrator 2021.

## Supplementary Material

Web_Material_uhag202

## Data Availability

The data underlying this article will be shared on reasonable request to the corresponding author. The data have not yet been uploaded to public databases (e.g. NCBI) and will be uploaded in accordance with the relevant requirements of the manuscript submission and publication process.
